# Harnessing Waste Bread: From Potential Use in Microbial Growth and Enzyme Production to Techno-Economic Assessment

**DOI:** 10.3390/microorganisms13071571

**Published:** 2025-07-03

**Authors:** Sameh Ben Mabrouk, Bouthaina Ben Hadj Hmida, Wejdene Sallami, Salma Dhaouadi, Theodoros Varzakas, Slim Smaoui

**Affiliations:** 1 Laboratory of Biochemistry and Enzymatic Engineering of Lipases, National Engineering School of Sfax, University of Sfax, BP 1173, Sfax 3038, Tunisia; benm_sameh@yahoo.fr (S.B.M.); bouthaina.benhadjhmidaa@gmail.com (B.B.H.H.); sellamiwejden22@gmail.com (W.S.); salmadhaouadidh@gmail.com (S.D.); 2 Department of Food Science and Technology, University of the Peloponnese, Antikalamos, 24100 Kalamata, Greece; 3 Laboratory of Microbial Biotechnology and Enzymatic Engineering (LMBEE), Centre of Biotechnology of Sfax (CBS), University of Sfax, Road Sidi Mansour Km 6, P.O. Box 1177, Sfax 3018, Tunisia; slim.smaoui@cbs.rnrt.tn

**Keywords:** waste bread, microorganisms, *Bacillus*, amylase, techno-economic assessment

## Abstract

This study highlights waste bread (WB) as a novel, cost-effective, and nutrient-rich substrate for microbial growth, offering a sustainable alternative to conventional media. As a renewable resource, WB promotes the circular economy by reducing food waste and encouraging biotechnological innovation. The incorporation of WB into microbial culture media enhanced the growth of various reference strains (*E. coli*, *E. faecalis*, *P. aeruginosa*, and *S. aureus*), with at least a two-fold increase compared to conventional Luria-Bertani (LB) medium. Moreover, combining 2% WB with diluted LB (1/10) reduced medium costs by up to 90%. Furthermore, it was confirmed that 1% WB can effectively replace starch during the screening of amylolytic strains. Applying a fractional factorial design, the production of amylase by *Bacillus* sp. BSS (Amy-BSS) was enhanced 15-fold. An analysis of the Pareto diagram revealed that WB was the most significant factor. Additionally, Amy-BSS was applied to hydrolyze polysaccharides in WB, enabling the generation of high-value-added products in food processing. This hydrolysis process yielded 4.6 g/L of fermentable sugars from 1% WB. Evaluating the economic feasibility of WB valorization into value-added products elucidates potential pathways for cost reduction and enhanced environmental sustainability, thereby positioning WB as a viable tool for sustainable development.

## 1. Introduction

In recent years, food systems have faced challenges due to climate change, rapid human population growth, accelerating urbanization, and epidemic surges. As a result, global food security gained significant priority from scientific communities worldwide [1,2]. One of the most critical obstacles to global food security is food waste. In this line, current studies revealed that roughly 33% of total food production, corresponding to 1.3 billion tons, is discarded every year. Notably, 14% of food loss arises during post-harvest and distribution phases, while 17% is wasted through retail and consumption, representing TND 750 billion/year in economic losses [3,4].

Owing to its perishable nature, (3–6 days shelf life), bread is the most frequently thrown-away food. Globally, ≈10% equivalent to 900.000 tons of bread is wasted across the entire supply chain, including production, transport, and consumption [5,6]. Meanwhile, the high demand for newly made bread exacerbates its overproduction, generating daily waste [7]. Bread’s rich nutrient composition could contribute to rapid quality loss, induced by chemical, physical, and especially mold deterioration. Additionally, during the baking, the starch is converted into a digestible gelatinized, susceptible to fungal attack [8,9].

In Tunisia, bread is a staple food and consumed daily. According to the National Institute of Consumption (INC), 42 kg of bread is wasted per household, amounting to around 113,000 tons of Tunisian bread being discarded annually [10]. In fact, a study on “Bread consumption by Tunisian families” proved that over 900,000 loaves are thrown away daily, with an estimated cost of TND 50 million, which affects the country’s economy [11].

Current bread waste disposal management approaches, such as incineration, landfilling, and anaerobic digestion, could pose diverse environmental issues, like soil contamination and generating greenhouse gases. Further, incineration could provoke air pollution through toxic emissions, odors, and disease vectors [12]. In response to these environmental challenges, nations admit an urgent necessity to convert to a zero-waste strategy by 2025. Therefore, developing waste valorization technologies is essential as a core component of sustainable waste management, while concurrently improving economic rationality [13]. In this context, innovative solutions have been investigated, including transforming stale bread into succinic acid [14], animal feed [15], 2,3-butanediol (BDO) [16], bioethanol [2], lactic acid [17], brewing adjunct for craft beer [18], and compost to enrich soil health [19]. Beyond these applications, efforts have focused on leveraging the nutritional profile of waste bread (WB), which consists of approximately 10% protein and 70% carbohydrates. As an upcycled substrate, these substances perform a valued reserve for microbial processes and enzymatic synthesis, which could enhance the efficiency and eco-friendliness of biotechnological claims [20]. Montemurro et al. [21] used WB as substrate to generate polyhydroxyalkanoates by *Haloferax mediterranei*. Similarly, Benabda et al. [22] employed WB to enhance the microbial proliferation of *Rhizopus oryzae* for the amylase and protease synthesis. Additionally, Jung et al. [23] utilized the glucose derived from the WB enzymatic hydrolysis to the *Euglena gracilis* cultivation of microalga, leading to the production of paramylon, which is valued in medicine and cosmetics sectors. In addition to these diverse applications, the food-processing industry provides openings to repurpose WB within the framework of a circular economy. For bakeries, using cost-effective wheat bran (WB) not only helps maintain competitive product pricing but also eliminates disposal costs. Additionally, they can generate extra revenue by selling leftover loaf waste [4]. For instance, Guerra-Oliveira et al. [24] studied the conversion of WB to make sugar-snap cookies. Furthermore, WB can be transformed into sugar hydrolysate to replace sucrose in the sweeteners and confections, to be used as bulking agents in bakery products, or to be processed into fructose syrup [15,25]. In addition, Immonem et al. [8] revealed that WB slurry increases the viscoelasticity of the dough, decreases the water absorption by 13%, and obtains less hard bread compared to the control.

Aligned with a zero-waste industrial strategy, this study investigates the valorization of Tunisian WB through four distinct applications: (1) as an alternative feedstock to replace conventional culture media for selected bacterial strains; (2) as a substitute for commercial starch in screening amylolytic microorganisms; (3) as a fermentation substrate to enhance amylase production; and (4) for the generation of sugar syrup via enzymatic hydrolysis using amylase (Amy-BSS).

## 2. Materials and Methods

### 2.1. Waste Bread Preparation and Characterization

The waste bread used in this study was prepared from white wheat flour, using *Saccharomyces cerevisiae* fermentation, following standard French bread-making protocols involving mixing wheat flour, salt, and yeast (https://www.inbp.com/). WB was collected from household food waste and local bakeries (Sfax government) after its shelf-life had expired or in the case of returns. The discarded bread exhibited no indications of mold, was sliced into roughly 1 cm^3^ pieces, and was left to dry at room temperature for four days. Afterwards, the slices were blended into a fine powder, using a laboratory blender, and stored at 4 °C until needed. The nutritional composition of the WB was assessed using analytical techniques. The moisture level was evaluated with a KERN DAB desiccator (Kern & Sohn, Balingen, Germany), while the ash content was performed via combustion in a muffle furnace (FLLI GALLI G/P model (Fratelli Galli, Fizzonasco, Italy)). Water activity was determined using a Rotronic RO-HP23-AW meter (Instrumat AG, Renens, Switzerland). The lipids, starch, and proteins were analyzed using MPA FT-NIR spectroscopy (Bruker, Billerica, MA, USA). The results are expressed as a percentage of dry matter.

### 2.2. Microorganisms, Medium Preparation, and Growth Conditions

Reference microorganisms, namely *Escherichia coli* DH5α, *Enterococcus faecalis*, *Pseudomonas aeruginosa*, and *Staphylococcus aureus*, were chosen for their ability to grow in conventional Luria-Bertani (LB) medium. Their proliferation was tested and compared in both conventional LB (C.LB) and non-conventional media (NC1 and NC2):

1. C.LB (g/L): 10 soya peptone, 5 yeast extract, 5 NaCl;
2. NC1 (g/L): 1 soya peptone, 0.5 yeast extract 0.5 NaCl, 20 g waste bread;
3. NC2 (g/L): 20 g waste bread with no additional nutrients.

A 14 h pre-culture of each microorganism was prepared in C.LB and used to inoculate the test media (NC1 and NC2) in 100 mL Erlenmeyer flasks, at an initial optical density (OD) of 0.4, for 24 h, at 37 °C and 200 rpm. Bacterial growth was monitored by measuring the OD at 600 nm, using a spectrophotometer. WB supernatants were used as blank for NC1 and NC2 fermentation media.

BSS strain isolated from a Tunisian biotope was used to produce amylase. Preliminary biochemical tests confirmed its classification as *Bacillus* species, hence its designation as *Bacillus* spp. BSS. The pre-culture was performed in basic LBS medium (C.LB + 1% soluble starch) at 30 °C, overnight, at 180 rpm. The bacterial cultures (OD = 0.2) were conducted following the experimental matrix (16 experiments with 1 central point) and incubated for 19 h at 30 °C in a rotary shaker.

Four amylolytic *Bacillus* strains (B1–B4) and *E. coli* DH5α (serving as negative control) were streaked in duplicate on solid WB medium (SWB), forming by C.LB +1% WB+ 1.8% agar), and incubated overnight at 37 °C. Following the incubation period, the plated samples were treated with 1% Lugol iodine solution (1 g I2 + 2 g KI) for 10 min. The presence of starch hydrolysis zones indicated amylase production [26]. A positive control (plate containing LBS medium) was used to verify the effectiveness of waste bread as a starch substitute for amylase-activity screening.

### 2.3. Crude Enzyme Preparation

The *Bacillus* spp. BSS strain grew in the optimized medium at 30 °C and 160 rpm for 14 h. The cells were harvested by centrifugation at 8000× *g* for 15 min. The resulting supernatant constituted the crude enzyme extract and exhibited an amylase activity of approximately 8 U/mL, using the optimized medium.

### 2.4. Amylase Activity Assay

Amylase activity of *Bacillus* spp. BSS was assessed using the 3,5-dinitrosalicylic acid (DNS). The reaction mixture consisted of 0.5 mL of 1% starch (Sigma (St. Louis, MO, USA)), 0.45 mL of phosphate buffer (pH = 7; 0.1 M), and 0.05 mL of crude enzyme, and it was kept at 60 °C for 10 min. After incubation, 1.5 mL of DNS was added to stop the enzymatic reaction. The mixture was boiled for 10 min, and 15 mL of deionized water was introduced. An independent control was prepared by mixing the substrate and DNS solution, followed by the enzyme, to remove all reducing sugars from the fermentation. The production of reduced sugars was determined at 550 nm. One unit of amylase activity is defined as the amount of enzyme that releases 1 µmol of reducing sugar per minute under the specified assay conditions. The α-amylase activity was measured as follows:

(1) $$\alpha \text{-}\mathrm{amylase}\;\mathrm{activity}\;(U/\mathrm{mL}/\min)=\frac{K\times OD}{180}\times 10^{-3}\times 10^{6}\times \frac{1}{10}\times \frac{1}{0.05}$$

where the molecular weight of glucose = 180; and K = the conversion coefficient of the DNS, which is 1.8.

### 2.5. Waste Bread Enzymatic Hydrolysis Assay

Enzymatic hydrolysis of WB was conducted using crude amylase produced from *Bacillus* spp. BSS. A total of 9 experiments were carried out following a designed matrix (Table 1). All experiments were carried out in 100 mL Erlenmeyer flasks that contained 20 mL of the various bread suspensions mixed with the enzymatic solution. After each experimental run, samples were collected and centrifuged at 8000× *g* for 15 min. The reducing sugars present in 1 mL of the hydrolyzed waste bread supernatant were measured by the DNS method, with glucose serving as the standard.

### 2.6. Fractional Factorial Design (FFD)

The $2_{V}^{k-p}$ fractional factorial plan designed with Minitab version 2019 (64-bit, LLC Minitab (State College, PA, USA) was chosen due to its simplicity, as it requires fewer experiments compared to the other designs. Thus, it can conserve both time and resources. In this study, a FFD $2_{V}^{5-1}$ was investigated for enhancing amylase production by *Bacillus* spp. BSS (y1) and the $2_{V}^{4-1}$ plan for evaluating the hydrolysis yield of WB using Amy-BSS (y2). The respective experimental variables for each response (y1 and y2) are detailed in Table 1 and Table 2.

The fractional factorial design led to the derivation of the subsequent empirical equation [27]:

(2) $$y=\beta_{0}+\sum_{i=1}^{k}\beta_{i}x_{i}\sum \sum_{j<i}\beta_{ij}x_{i}x_{j}+\varepsilon \;$$

where *β*_0_, *β_i_*, and *β_ij_* are the regression coefficients for the constant, linear, and interaction coefficients; *y* is the response; *x_i_* and *x_j_* are the independent variables; and *ε* is the experimental error. In FFD, the interaction effects among three or more variables are presumed to be inconsequential.

### 2.7. Statistical Analysis and Model Adequacy

The adequacy of the regression model was assessed through multiple statistical parameters. Analysis of variance (ANOVA) with Fisher’s test (F-value) assessed the overall model significance. Individual variable relevance and bi-interactions were evaluated using *p*-value and Student’s test (*t*-test) at a 95% confidence range. The coefficient of determination (R^2^), along with the predicted and adjusted R^2^ values, indicates the model’s goodness of fit—where values closer to 100% reflect greater predictive accuracy. The experimental designs were carried out in consistent conditions within a single block of measurements, and the sequence of the experiments was kept non-randomized to reduce the impact of uncontrolled variables. All the experiments were conducted in n = 3 ± standard deviation (SD).

### 2.8. Material Cost Calculation (Techno-Economic Assessment)

The available price on the Sigma Aldrich website (accessed on 16 April 2025) was used to estimate the cost of additional ingredients for media. Peptone, yeast extract, sodium chloride (NaCl), and starch were used to prepare CLB, NC1, and LBS media. The cost assessment was derived by computing the required material quantity, as detailed below:

(3) $$Cost\;per\;unit\;\left(€\right)=\frac{material\;cost\left(€\right)}{100\left(g\right)}\times material\;used\;\left(g\right)$$

(4) $$Total\;cost\;\left(€\right)=\Sigma \;cost\;per\;unit\;(€)$$

(5) $$Percentage\;price\;decrease\;\left(\%\right)=\frac{\left(original\;price-new\;price\right)}{originalprice}\times 100$$

### 2.9. High-Performance Liquid Chromatography (HPLC)

The products of starch hydrolysis from waste bread (1%) after 1 h, 2 h, and 3 h of hydrolysis were analyzed by high-performance liquid chromatography (HPLC Agilent 1100 (Agilent, Santa Clara, CA, USA)). The reactions were stopped by boiling the samples for 5 min, filtered through a 0.22 mm membrane, and applied to the HPLC system carbohydrate column (Shodex sugar KS-802H901093 (Shodex, Tokyo, Japan); 8 mm × 300 mm). Isocratic water was the mobile phase with an elution rate of 1 mL per min throughout the analysis. Detection was performed using the retractive index detector (Waters RI 401, profcontrol.de, Schönwalde-Glien, Germany). The hydrolysis products were assigned by comparison with standard glucose, maltose, and unhydrolyzed WB.

## 3. Results

### 3.1. Waste Bread Characterization

The proximate analysis of the waste bread is summarized in Table 3. The WB showed moisture content of 17.17% and water activity of 0.761. Starch is the dominant component (61.41%), with an intermediate protein quantity (8.88%). These characteristics highlight the significant bread’s potential as a feedstock for microbial fermentation, enzymatic hydrolysis, and nutrient recovery.

### 3.2. Waste Bread for Bacterial Growth

**Figure 1:**
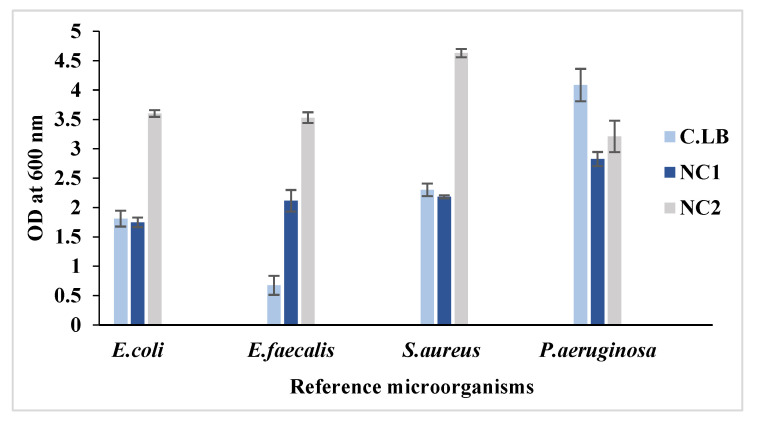
Bacterial growth of reference microorganisms in different culture media (C.LB, NC1, and NC2).

In this study, two types of bread waste-based media (NC1 and NC2) were formulated to test bacterial proliferation. Microbial growth was investigated in diluted culture media. In Figure 1, it can be observed that *E. coli* displayed a two-fold increase in NC2 medium (3.6) compared to CLB (1.81). *E. faecalis* demonstrated a five-fold enhancement in NC2 (3.53 vs. 0.675 in CLB), while *S. aureus* exhibited the highest growth rate in NC2 (4.63), surpassing its growth in both CLB and NC1. Though *P. aeruginosa* thrived best in CLB (4.085), it also showed considerable growth in WB (OD 3.21). These results imply that WB effectively meets the nutritional needs of various bacterial strains, making it a promising alternative culture medium. Notably, the NC1 formulation resulted in only modest growth rates for all microorganisms tested.

### 3.3. Waste Bread for Amylolytic Strains Screening

**Figure 2:**
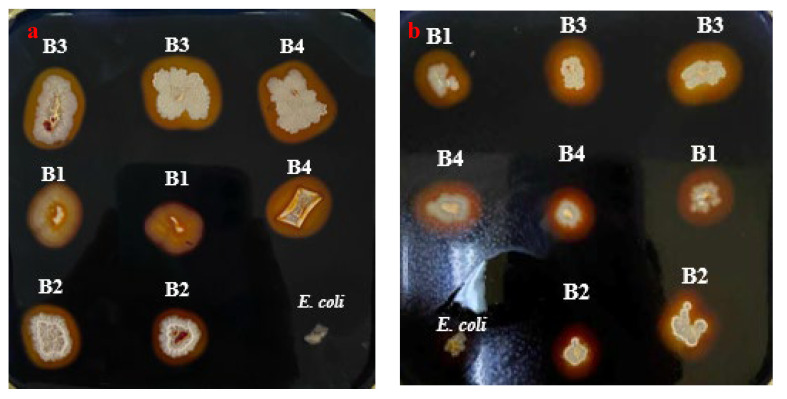
Screening assay of amylolytic strains after incubation at 37 °C for overnight. (**a**) *Bacillus* strains incubated on SWB plate. (**b**) Bacterial colonies on LBS plate.

To assess the amylolytic potential of *Bacillus* strains (B1–B4), waste bread plates (SWB) were employed as the only supplier of starch in the screening assay. After an overnight incubation at 37 °C, plates were flooded with iodine solution, revealing various transparent halos around positive bacterial colonies, indicative of starch hydrolysis (Figure 2a,b). However, quantitative analysis demonstrates a relevant difference in enzymatic activity between SWB and LBS plates. Notably, hydrolysis-zone diameters in the LBS plate were larger for all the *Bacillus* strains (B1: 0.55, B2: 0.45, B3: 0.7, and B4: 0.65 cm), respectively, against (0.25, 0.35, 0.6, and 0.55 cm) in the SWB plate.

These variations can be attributed to differences in substrate composition. Commercial LBS medium contains purified starch, whereas the SWB medium relies on the starch content naturally present in the bread waste matrix and the amylaceous fraction of the flour used. Furthermore, the reduction in hydrolysis halo size between LBS and SWB was 54.5%, 22.2%, 14.3%, and 15.4% for B1, B2, B3, and B4, respectively. These reductions ranging from ≈14 to 55% indicate that LBS is a more sensitive medium. However, despite the smaller halo zones observed with SWB, it maintained full detection reliability, showing 100% concordance in identifying positive amylolytic strains. Notably, the cost of preparing SWB was approximately 85% lower than that of LBS, offering a significant economic advantage for large-scale screenings. Despite these variations, using waste bread as a substrate for amylolytic-strain screening preserved detection sensitivity, as the patterns of clearance zones stayed like those seen in commercial starch agar.

### 3.4. Fractional Factorial Design (FFD)

#### 3.4.1. The $2_{V}^{5-1}$ for the Optimization of Amylase Production

The measured response of the Amy-BSS activities is indicated in Table 4. Amylase activity varied depending on the fermentation conditions, ranging from 3.76 U/mL to 8.75 U/mL. The highest enzymatic activity was achieved in trials 1 and 6, with 8.75 U/mL and 8.78 U/mL, respectively. It is worth noting that the initial activity of the Amy-BSS in non-optimized medium (conventional LBS) was recorded as 0.69 U/mL. An improvement of ≈13-fold proves the efficiency of this experimental plan.

To determine the significant effect of the main variables and their two-way interactions, ANOVA was performed. The significance of variables was evaluated by the small *p*-value < 0.05, indicating high significance. From Table 5, all the main factors are significant, having the same *p*-value < 0.05, except for the NaCl, which had a *p*-value of 0.46 > 0.05. Conversely, yeast extracts (A), soya peptone (B), waste bread (D), and wheat starch (E) showed different *F*-values of 46.79, 70.21, 131.72, and 27.03, respectively.

**Figure 3:**
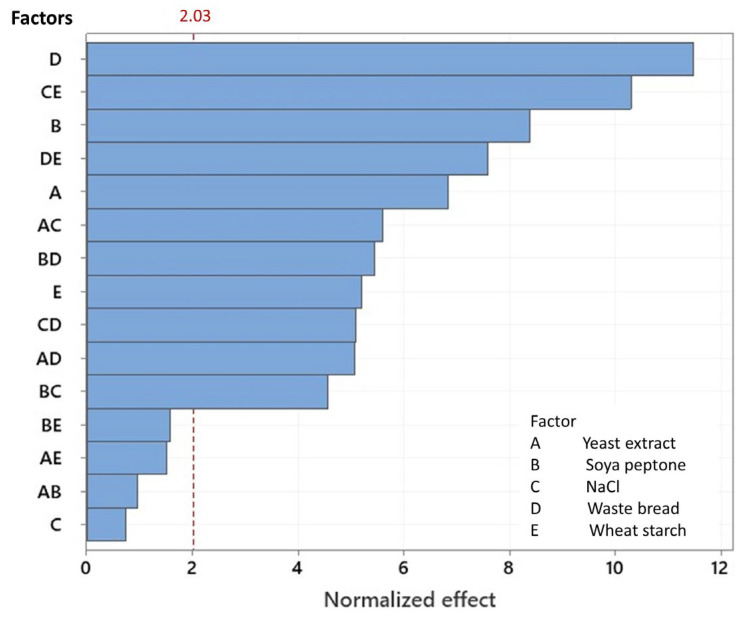
Standard Pareto chart of the normalized effect for the Amy-BSS production.

This enables the use of a Pareto chart to visually analyze the impact of the most influential factor. Hence, the Pareto chart (Figure 3) clearly revealed the high influence of WB on the improvement of Amy-BSS production following the addition of soya peptone (A), yeast extract (B), and wheat starch (E). WB had the greatest effect on enzyme production owing to its high starch content, which acts as a highly effective inducer of amylase production, although NaCl (C) itself was not significant. In contrast, its interaction with yeast extract (A × C), soya peptone (B × C), waste bread (D × C), and wheat starch (E × C) significantly affected the enzymatic production. Thus, these interactions were ranked according to their *F*-values, as follows: (C × E), (A × C), (C × D), and (B × C), with *F*-values of 105.83 > 31.29 > 25.86 > 20.82, respectively (Table 5), suggesting their substantial contribution to the response (y_1_).

Additionally, the plot indicates that all data points are normally distributed around a mean of zero and adhere to a straight line.

The mathematical model corresponding to this design is given by the following equation:


y1 = 5.61 + 2.36 A − 4.79 B + 3.08 C + 0.666 D + 7.75 E + 1.01 A × B + 5.86 B × C − 2.658 B × D + 1.6 A × E − 4.78 B × C +2.852 B × D − 1.66 B × E + 2.665 C × D − 10.78 C × E − 0.823

The goodness of fit was evaluated using ANOVA, which yielded an F-value of 36.75 and a *p*-value < 0.05. Moreover, the coefficient of determination, R^2^, was equal to 0.9453, meaning that 94.53% of the observed variation is attributed to the variable effects. Thus, it can be concluded that the predicted model (R^2^pred = 87.7%) fits well with the experimental data (R^2^adj = 91.96%). This set of results clearly demonstrates the validity of the model.

**Figure 4:**
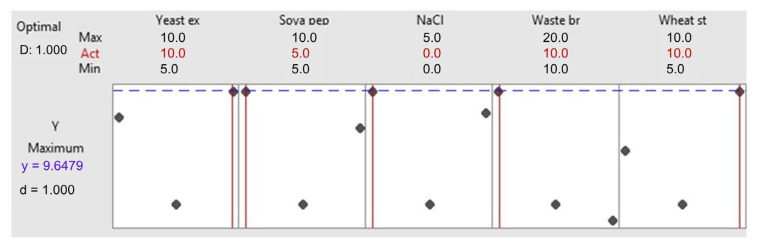
Optimal factors setting for Amy-BSS production. Blue line represents the predicted maximum response. Red vertical lines indicate the optimal levels of each factor used in the solution; Black squares correspond to experimental design points.

Figure 4 shows the optimized levels for the chosen variables. It is observed that the ideal values for yeast extract, soya peptone, NaCl, waste bread, and wheat starch are 10, 5, 0, 10, and 10 g/L, respectively, achieving an amylase activity of 9.64 U/mL with a desirability of d = 1. To ensure the reproducibility of this composition, three independent experiments were executed. An amylase activity of 8.96 ± 0.32 U/mL was obtained, close to the predicted value, confirming the reliability of the method.

#### 3.4.2. The $2_{V}^{4-1}$ for the Enzymatic Waste Bread Hydrolysis

An optimal condition for enzymatic hydrolysis of WB by Amy-BSS was defined by $2_{V}^{4-1}$ fractional factorial design. Four parameters, amylolytic activity (X_1_), incubation temperature (X_2_), waste bread concentration (X_3_), and hydrolysis time (X_4_), were varied at two different levels to investigate their effects and interactions on reducing sugar yield. Table 6 groups the measured experimental responses corresponding to the eight tests runs. The highest amount of released sugar was 4.635 g/L, obtained in run 2 using 10 U of Amy-BSS, a 50 °C incubation temperature, and 1% WB during 3 h of hydrolysis time.

**Figure 5:**
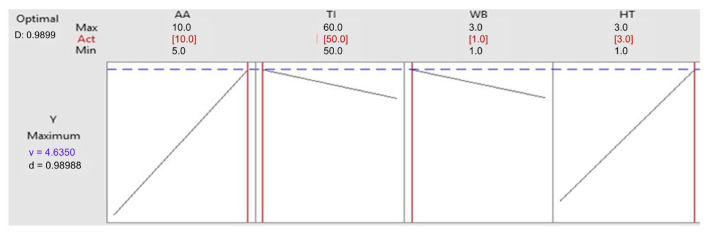
Optimized response design and data responses resulting for the $2_{V}^{4-1}$ plan. AA, amylase activity; TI, temperature of incubation; WB, waste bread; HT, hydrolysis time. Blue line represents the predicted maximum response; Red vertical lines indicate the optimal levels of each factor used in the solution.

This result was confirmed to be optimal, as demonstrated by Figure 5, which shows the highest reducing sugar yield under this condition.

The statistical significance of each individual factor and their combinations at α = 5% was evaluated using ANOVA. As shown in Table 7, the main (X_1_, X_2_, X_3_, and X_4_) and interaction effects, (X_1_ × X_2_), (X_1_ × X_3_), and (X_1_ × X_4_), are statistically significant (*p* << 0.05).

A first-order polynomial model was used to describe the correlation between the four tested variables and the hydrolysis yield. The fit of the data is described as follows:


y2 = −0.367 +0.4320 X_1_ + 0.00575 X_2_ + 0.60701 X_3_ − 0.3221 X_4_ − 0.005583 (X_1_ × X_2_) − 0.08525 (X_1_ × X_3_) + 0.14658 (X_1_ × X_4_).

The analysis of the model equation reveals that waste bread (X_3_) exerts the strongest positive effect on y_2_ (+0.60701), while hydrolysis time (X_4_) had a notable negative impact (−0.3221), making them the main drivers of change in y_2_. Hence, X_3_ acts as a key promoter, and X_4_ as an inhibitor. On the contrary, X_2_ shows a negligible effect (+0.00575), and X_1_ displays a moderate positive influence (+0.4320). Additionally, the antagonistic interactions (X_1_ × X_2_) and (X_1_ × X_3_) suggest that high levels of these variables reduce the effectiveness of X_1_ due to temperature sensitivity and limited enzyme accessibility. Meanwhile, the synergistic interaction (X_1_ × X_4_) (+0.146) indicates that extended hydrolysis time enhances the X_1_ positive impact on y_2_ through improved substrate accessibility and sustained enzyme activity.

**Figure 6:**
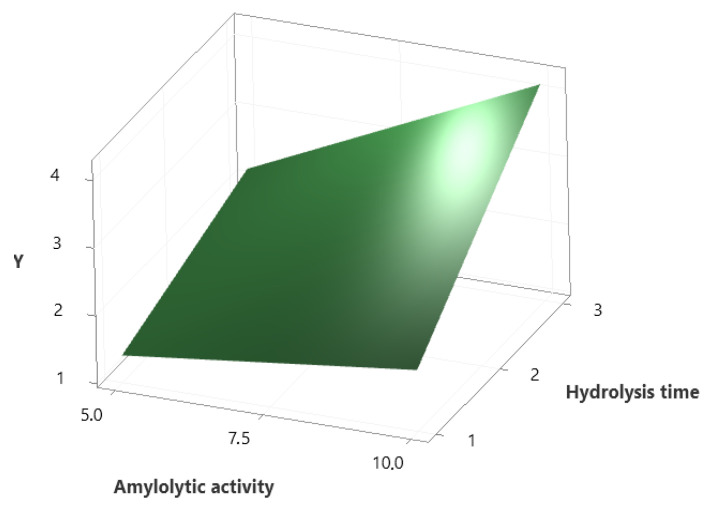
The surface plot of the synergistic interaction (amylolytic activity × hydrolysis time). y is the hydrolysis yield.

The synergistic interaction and its significant effect are further illustrated by the surface plot in Figure 6. The plot reveals that the highest hydrolysis yield occurred on the top-right corner, corresponding to the combination of high Amy-BSS concentration (10 U) and extended hydrolysis time (3 h). This trend aligns with enzymatic action: at low amylase concentrations, the reaction rate is limited by the scarcity of active sites for starch binding, increasing both the enzyme load and duration-maximized substrate conversion. These results experimentally validate the (X_1_ × X_4_) synergy identified in the model equation, demonstrating how optimized enzyme–time enhanced hydrolysis yield.

The validity of the adjusted model was assessed based on the results in Table 7. The regression analysis indicates that the model is highly significant, with a very low *p*-value (*p* < 0.05) and a high F-value (1226.51).

Meanwhile, the coefficient of determination (R^2^ = 99.83%) indicates that only 0.17% of the total variability was not explained by the model, suggesting that the considered variables were sufficient to make reliable predictions. Additionally, the small difference between the adjusted R^2^ (99.74%) and the estimated R^2^ (99.59%) demonstrates a strong correlation between the estimated and experimental data.

### 3.5. High-Performance Liquid Chromatography (HPLC)

**Figure 7:**
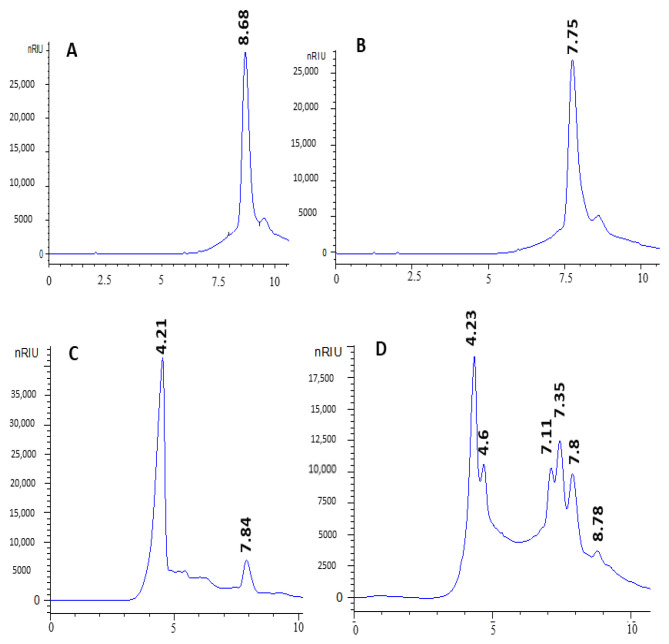
Hydrolysis products of 3% WB using 10U of Amy-BSS: (**A**) glucose solution of 5 mg/mL, (**B**) maltose solution of 5 mg/mL, (**C**) 3% unhydrolyzed WB, and (**D**) hydrolyzed WB by Amy-BSS.

HPLC analysis revealed that glucose, and other sugars with a degree of polymerization (DP) ≥ 3 were the final products of WB starch (Figure 7). It appears that the amylase generated by *Bacillus* spp. BSS is an endo-amylase. Indeed, its action on WB starch primarily produces oligosaccharides with a DP ≥ 3, along with a fraction of glucose and maltose. Conversely, exoamylases act in a progressive manner from the non-reducing ends of the polysaccharide chain, resulting in the sequential release of only maltose or glucose.

## 4. Discussion

The growing global population has led to an increase in food quantities, resulting in a surge in food waste (FW). This is part of the aftermath of an absolute linear economy model. Recently, the circular economy has gained traction as a sustainable alternative. It enables FW recycling in the industrial process via new technologies. This approach aligns with the concept of “cradle to cradle”, where the recycled materials retain their features and can be re-introduced to the environment without harm [28]. Bread ranks as the most discarded food waste globally due to its rapid spoilage. Its valorization through multiple biological approaches offers a promising pathway for promoting a circular economy.

To develop effective valorization approaches, a comprehensive waste stream analysis is required. Hence, the characterization study (Table 3) showed that WB contained 61.41% starch, which is in accordance with Kumar et al. [4], who reported a content of 50–70%. Additionally, the moisture content (17.17%) and water activity (0.761) are critical parameters affecting bread stability. In food processing, moisture content influences preservation, storage, packaging, and transportation. Thus, the high level of moisture leads to mold growth within 3–7 days. Our outcomes are in close agreement with the findings of Gobbetti et al. [29], who reported a water activity of about 0.97. Notably, the ash quantity, 0.64%, was significantly lower than that observed by Abidin et al., i.e., 6.74% [30].

Given its rich starch content and nutrient profile, WB serves as an attractive low-cost substrate for fermentation [31]. In general, bacterial cultivations are achieved successfully using conventional medium, such as Luria-Bertani broth (LB). However, LB is expensive due to the elevated-cost components, such as yeast extract, soya peptone, and beef extract, which are the basic nitrogen additives [13]. The development of an alternative growth media using WB aims to strike a balance between where we can obtain a cost-effective medium with good biomass yields. The used strains (*E. coli*, *E. faecalis*, *S. aureus*, and *P. aeruginosa*) were routinely cultured in LB. Interestingly, our results have shown superior microbial growth in the novel formulated media (NC1 and NC2) compared to LB.

The variations observed in the growth of the pathogenic microorganisms using the waste bread media (NC1 and NC2) can be explained by the difference in the metabolic process and nutrient preferences of each strain. In other words, the improved growth of *E. coli*, *E. faecalis*, and *S. aureus* in NC2 could be attributed to their ability to consume waste bread and satisfy their need to achieve better growth, which likely explains their superior growth in NC2 compared to the control. Meanwhile, *P. aeruginosa* may prefer more complex and pure nitrogen and carbon sources (commercial), even its demonstrated viable proliferation. Hence, the differential growth patterns could be influenced by the ability of each microorganism to metabolize a specific nutrient released from bread waste. This result validates WB as a cheaper nitrogen source for microbial cultivation.

Our findings are in concordance with Verni et al. [5], who confirm WB’s efficacy as a nutrient rich growth medium for starter yeasts and fungi, surpassing classical media. Meanwhile, lactic acid bacteria [13] and fungi [32] showed significant microbial growth on the WB-based medium. In addition, the effective cultivation of these strains on WB-based media (NC1 and NC2) confirms the ability of the media to support microbial growth under conditions relevant to food microbiology. This indicates that WB is suitable for potential applications in food safety monitoring and environmental pathogen screening. Conversely, some researchers have used enzymatic pretreatment of WB before it was incorporated into the fermentation media. For instance, Carsanba et al. [33] treated WB with amylase, glucoamylase, and protease before its use to cultivate *Yarrowia lipolytica* strain K57. However, such pretreatments increase the overall cost of the fermentation process, counteracting the objective of waste valorization for cost reduction. Above all, when comparing CLB with NC1, we showed that the costs of the fermentation medium could be reduced by 90%, and with NC2, the expenses could be eliminated completely. Additionally, expired bread can successfully substitute pure starch for screening amylolytic bacteria, as was previously shown. Furthermore, using SWB instead of LBS reduced costs by 46.68% (Table 8).

Traditionally, α-amylase production has relied on submerged fermentation with starch as the sole carbon and energy source. However, this conventional source faces significant economic challenges, as the cost of the carbon source constitutes a major portion of amylase production expenses. This restriction often makes the process unfeasible economically [31]. To address this challenge, exploring agro-waste as a substitute substrate is one of the solutions. These wastes naturally occurred with carbon storage, acting as an energy source throughout microbial fermentation [34]. This results in a promising option for economical industrial amylase production. The use of agricultural waste, such as moong husk, soybean cake, rice bran, and groundnut shell, as media supplements in α-amylase production for *Bacillus amyloliquefaciens* KCP2, *Bacillus tequilensis* TB5, *Aspergillus*, respectively, has been reported [35,36,37]. Recently, WB has sparked interest as a promising fermentation feedstock for enzymatic synthesis due to its richness in carbohydrates, calories, proteins, and essential vitamins [38]. Its nutritional profile has been investigated in this study as an ideal energy and carbon source for Amy-BSS production.

To further optimize and reduce the cost of amylase production, a fractional factorial design (FFD) was employed in this study to systematically evaluate the effects of key process variables. Unlike full factorial or response surface designs, FFD requires fewer experimental runs while still revealing valuable insights into key variables. When executing 17 runs (Table 4), it has been found that WB significantly improved Amy-BSS production from 0.69 U/mL to 8.96 U/mL. This enhancement confirms that WB is a valuable carbon/substrate source. Furthermore, based on the optimized response (Figure 5), it was demonstrated that a low level of soy peptone was sufficient, reducing the required amount of nitrogen sources thanks to the compensatory effect of WB. Additionally, the absence of NaCl needed for Amy-BSS production suggests that *Bacillus* BSS’s sodium requirement was met by the inherent salt quantity present in the WB. Moreover, bread contains the key amino acids (nicotinic acid, thiamine, biotin, and pantothenic acids), as reported by Abd-Elhalim et al. [39]. These elements support *Bacillus* spp. BSS growth while minimizing dependence on synthetic amino acid supplementation. The re-purpose of WB lowers Amy-BSS production cost, further preserving bacterial biomass yield. A major advantage of WB is its non-seasonal availability, unlike conventional agro-residues, facilitating consistent integration into circular economy systems.

Alternatively, in order to apply waste bread in food processing, its modification through hydrolysis is crucial. Hydrolysis breaks the complex configuration of WB starch into fermentable sugars that can be easily converted into valuable food supplements. As opposed to lignocellulosic waste, WB is a more efficient source of fermentative sugars due to its high starch quantity [33]. Notably, sugar generated from WB is without inhibitors, commonly found in lignocellulosic compounds such as furans, organic acids, and phenols, which may reduce the activity of hydrolytic enzymes [2,40]. Nevertheless, the starch WB can be hydrolyzed using enzymatic or acid hydrolysis. Commonly, dilute acid hydrolysis through either HCl or H_2_SO_4_ has been applied to cleave glycosidic bonds, yielding lower-molecular-weight carbohydrates [41]. However, chemical hydrolysis demands excessive energy input, corrosive substances, and severe operating settings, which can generate harmful byproducts. In contrast, enzymatic pretreatment offers a more sustainable and eco-friendly approach, leveraging the high specificity and efficiency of enzymes to cleave starch under mild conditions (40–50 °C). Therefore, enzymatic hydrolysis consumes and hydrolyzes with no toxic substance compared to the chemical approach [17,42].

Commercial enzymes, namely α-amylase (Grindamyl A14000), amyloglucosidase (Grindamyl PlusSweet), maltogenic amylase (MALT), and protease (Corolase 7089), were investigated for the enzymatic hydrolysis of WB, as reported by Rosa-Sibakov et al. [15]. Yet, to make an economical process, substituting commercial enzymes with house laboratory enzymes is crucial. Therefore, we have prioritized the use of Amy-BSS for this purpose. As evidenced by FFD, the reducing sugar yield through WB hydrolysis was significantly dependent on hydrolysis time, Amy-BSS unities, WB ration, and incubation temperature. Our results proved that fermentable sugar production increased with longer time and higher Amy-BSS unities, in accordance with Abidin et al. [30], who found that the maximum reducing sugar levels are obtained at an enzyme concentration of 6% (*w*/*v*), as opposed to 2% and 4%. Additionally, when hydrolysis periods increase, the concentration of reducing sugars also increase, peaking at 180 min. In addition, Mihajlovski et al. [43] reported that hydrolysis yield was influenced by fermentation time and WB concentration. In contrast, in our findings, 1% of WB was optimal because higher substrate increased viscosity, thereby restricting Amy-BSS action. Further, incubation temperature (50°) showed a positive effect, which contradicts the Kahlouche et al. [44] results. They found that changing the temperature from 20 °C to 100 °C had no discernible impact on the hydrolysis of waste bread (*p*-value of 0.069 > 0.05). The main differences between the two studies could account for this discrepancy. Interestingly, our investigation used a bacterial α-amylase, while Kahlouche et al. [44] used a commercial α-amylase from a fungus source, *Aspergillus oryzae* (A8220-50ML, Sigma-Aldrich, Søborg, Denmark). There are notable physiological and biochemical distinctions between these two enzyme sources, which are from different kingdoms. Furthermore, to lower the economic cost of industrial-scale applications, we utilized a crude extract from a laboratory strain, non-commercial, even though their enzyme was refined and commercially produced.

As illustrated in Figure 7, the fermentable sugars produced through Amy-BSS activity were primarily glucose, along with sugars having a DP ≥ 3, demonstrating a promising potential for reuse in new bread-making applications. It has been demonstrated that certain strains, including *Bacillus* species and *Leuconostoc mesentroides*, may convert waste bread into mannitol, a sugar alcohol used in the food sector [45]. Mihajlovski et al. [43] identified maltose, glucose, and maltotriose as main sugars products generated during WB hydrolysis using enzymes from *Hymenobacter* sp. CKS3. In a nutshell, by replacing conventional sugar with enzymatically hydrolyzed WB syrup, the food industry, particularly bakeries, can significantly reduce production costs. These syrups can completely replace standard sugars in the making of bread and boost shelf-life through improved hygroscopicity and crumb tenderization, as well as flavor and crust color via Maillard reactions [15]. Such practical incorporation of bread waste-derived components offers bakeries a scalable solution to align production with both financial and environmental goals.

## 5. Conclusions

This study highlights the feasibility of valorizing Tunisian waste bread (WB) as a sustainable and cost-effective substrate for various biotechnological applications, supporting its role in enhancing circular economy. The formulation of WB-based culture media not only reduced production costs by up to 90% compared to conventional LB media but also significantly enhanced microbial growth, confirming WB’s economic and functional advantages. The use of just 1% WB as a replacement for commercial starch in amylolytic-strain screening further validated its applicability as a sustainable alternative. Moreover, the application of FFD led to a 15-fold increase in amylase production by the Amy-BSS strain, with WB identified as the most influential variable, directly supporting the aim of optimizing bioprocess efficiency. In addition, enzymatic hydrolysis of WB under mild conditions yielded 4.6 g/L of fermentable sugars, demonstrating its viability as a raw material for bio-based product development. Altogether, these outcomes confirm that Tunisian WB is a robust and efficient resource, aligning with the study’s goal of advancing sustainable waste valorization within a circular bioeconomy framework.

## Figures and Tables

**Table 1:** Variables and experimental domains in the WB hydrolysis ($2_{V}^{4-1}$).

Variables	Unity	Level −1	Level 1
X_1_: Amylolytic activity	Unities (U)	5	10
X_2_: Incubation temperature	°C	50	60
X_3_: Waste bread concentration	%	1	3
X_4_: Hydrolysis time	Hours (h)	1	3

**Table 2:** Levels of factors assessed in the optimization of amylase production ($2_{V}^{5-1}$).

Variables (g/L)	Level −1	Level 0 *	Level 1
A: Yeast extract	5	7.5	10
B: Soya peptone	5	7.5	10
C: NaCl	0	2.5	5
D: Waste bread	10	15	20
E: Wheat starch	5	7.5	10

* Level 0 is applied to enhance the prediction variance within the experiment and to offer an extra level for assessing potential lack of fit.

**Table 3:** Compositional analysis of Tunisian white bread waste.

Property	Quantity (%)
Moisture	17.17
Ash	0.64
Water activity	0.761
Lipids	2
Starch	61.41
Proteins	8.88

**Table 4:** Matrix of $2_{V}^{5-1}$ design in coded levels for amylase production with experimental values.

Run Order	Yeast Extract (A)	Soya Peptone (B)	NaCl (C)	Waste Bread (D)	Wheat Starch (E)	Experimental Amy-BSS Activity (U/mL)
1	−1	−1	−1	−1	1	8.75 ± 0.32
2	1	−1	−1	−1	−1	7.58 ± 0.53
3	−1	1	−1	−1	−1	5.95 ± 0.57
4	1	1	−1	−1	1	8.35 ± 0.36
5	−1	−1	1	−1	−1	7.53 ± 0.45
6	1	−1	1	−1	1	8.87 ± 0.36
7	−1	1	1	−1	1	3.76 ± 0.20
8	1	1	1	−1	−1	7.42 ± 0.28
9	−1	−1	−1	1	−1	5.85 ± 0.85
10	1	−1	−1	1	1	5.11 ± 0.60
11	−1	1	−1	1	1	5.41 ± 0.53
12	1	1	−1	1	−1	5.57 ± 0.36
13	−1	−1	1	1	1	4.62 ± 0.36
14	1	−1	1	1	−1	8.27 ± 0.50
15	−1	1	1	1	−1	6.73 ± 0.39
16	1	1	1	1	1	4.59 ± 0.42
17	0	0	0	0	0	5.70 ± 0.10

**Table 5:** Analysis of variance (ANOVA) for the $2_{V}^{5-1}$ plan.

Source	DL	F-Value	*p*-Value
Model	16	36.75	<<0.05
Main factors	5	55.26	<<0.05
Yeast extract (A)	1	46.79	<<0.05
Soya peptone (B)	1	70.21	<<0.05
NaCl (C)	1	0.56	0.46
Waste bread (D)	1	131.72	<<0.05
Wheat starch (E)	1	27.03	<<0.05
Interaction 2 factors	10	30.24	<<0.05
A × B	1	0.93	0.342
A × C	1	31.29	<<0.05
A × D	1	25.73	<<0.05
A × E	1	2.32	0.137
B × C	1	20.82	<<0.05
B × D	1	29.60	<<0.05
B × E	1	2.52	0.122
C × D	1	25.86	<<0.05
C × E	1	105.83	<<0.05
D × E	1	57.52	<<0.05

**Table 6:** Design and data responses resulting in the $2_{V}^{4-1}$ plan.

Run	Amylase Activity	Incubation Temperature	Waste Bread	Hydrolysis Time	Experimental Hydrolysis Yield (g/L)
1	−1	−1	−1	−1	1.277 ± 0.099
2	1	−1	−1	1	4.635 ± 0.049
3	−1	1	−1	1	1.877 ± 0.012
4	1	1	−1	−1	1.847 ± 0.015
5	−1	−1	1	1	2.460 ± 0.078
6	1	−1	1	−1	1.857 ± 0.051
7	−1	1	1	−1	1.417 ± 0.006
8	1	1	1	1	3.643 ± 0.023

**Table 7:** ANOVA for the released sugar by Amy-BSS.

Source Variation	DL	F-Value	*p*-Value
Model	7	1126.51	<<0.05
Main factors	4	1945.07	<<0.05
Amylolytic activity (X_1_)	1	3146.82	<<0.05
Incubation temperature (X_2_)	1	267.98	<<0.05
Waste bread (X_3_)	1	8.57	<<0.05
Hydrolysis time (X_4_)	1	4962.70	<<0.05
Interaction 2 factors	3	472.41	<<0.05
X_1_ × X_2_	1	40.01	<<0.05
X_1_ × X_3_	1	373.09	<<0.05
X_1_ × X_4_	1	1103.06	<<0.05

**Table 8:** Cost comparison of different culture media used.

Media	Media Components	Component Cost (EUR/100 g)	Component Used (g/L)	Cost per Unit (EUR)	Total Cost (EUR)
CLB	Peptone soya	28	10	2.8	5.14
	Yeast extract	33.68	5	1.68	
	NaCl	13.2	5	0.66	
NC_1_	Peptone soya	28	1	0.28	0.514
	Yeast extract	33.68	0.5	0.168	
	NaCl	13.2	0.5	0.066	
	Waste bread	0	10	0	
NC_2_	Waste bread	0	0	0	0
LBS	Peptone soya	28	10	2.8	9.64
	Yeast extract	33.68	5	1.68	
	NaCl	13.2	5	0.66	
	Soluble starch	45	10	4.5	

Take into consideration that EUR 1 = TND 3.34 (exchange rate as of 16 April 2025).

## Data Availability

The original contributions presented in this study are included in the article. Further inquiries can be directed to the corresponding author.
